# [Expression of Concern] Circular RNA ATAD1 is upregulated in acute myeloid leukemia and promotes cancer cell proliferation by downregulating miR-34b via promoter methylation 

**DOI:** 10.3892/ol.2026.15802

**Published:** 2026-08-05

**Authors:** Yarong Wu, Bingjun Gao, Xiaolei Qi, Liyun Bai, Bixin Li, Hongjing Bao, Xi Wu, Xiaoyun Wu, Yuxia Zhao

Oncol Lett 22: 799, 2021; DOI: 10.3892/ol.2021.13060

Following the publication of the above paper, it has been drawn to the Editor's attention by a concerned reader that, regarding the cellular images of the Kasumi-6 cells shown in Fig. 4D on p. 7, the ‘circ-ATAD1’ and ‘circ-ATAD1+miR-34b’ data panels are apparently matching, suggesting that this figure has been assembled incorrectly

The authors have been contacted by the Editorial Office to offer an explanation for this apparent anomaly in the presentation of the data in this paper, and we are awaiting their response. Owing to the fact that the Editorial Office has been made aware of this potential issue surrounding the scientific integrity of this paper, we are issuing an Expression of Concern to notify readers of this potential problem while the Editorial Office continues to investigate this matter further.

